# Association of a stellate ganglion block-based care pathway with hypnotic medication reduction in chronic insomnia with anxiety symptoms: a real-world retrospective cohort study

**DOI:** 10.3389/frsle.2026.1923070

**Published:** 2026-09-25

**Authors:** Dongze Wang, Xiaohan Wang, Wenyuan Wang, Yanyong Wang

**Affiliations:** Department of Neurology, The First Hospital of Hebei Medical University, Shijiazhuang, Hebei, China

**Keywords:** anxiety symptoms, benzodiazepines, chronic insomnia, hypnotic deprescribing, propensity score, real-world evidence, stellate ganglion block, Z-drugs

## Abstract

**Background:**

Chronic insomnia with anxiety symptoms is frequently accompanied by sustained hypnotic or sedative medication use. Evidence regarding procedure-based adjuncts to structured medication reduction remains limited.

**Objective:**

To evaluate whether participation in a stellate ganglion block (SGB)-based care pathway, compared with contemporaneous routine care without SGB, was associated with hypnotic or sedative medication reduction at 1 month.

**Methods:**

This single-center retrospective cohort study screened 420 records from 1 January 2022 to 31 December 2025 and included 160 adults with chronic insomnia, anxiety symptoms, and baseline hypnotic or sedative use. Eighty patients received a standardized four-session SGB-based pathway and 80 routine-care controls were selected by outcome-blinded, 1:1 calendar-time risk-set sampling without replacement. The pragmatic 1 month composite outcome was achievement of at least one of the following: a ≥50% reduction in the exploratory operational medication-burden score, transition from combination therapy to monotherapy, or complete discontinuation maintained for ≥2 weeks. Components and class-specific benzodiazepine and Z-drug outcomes were analyzed separately. The primary adjusted model was restricted to age, sex, baseline operational burden, and baseline medication count. Robust Poisson, Firth logistic, marginal standardization, bootstrap, propensity-score matching, overlap weighting, and trimmed stabilized inverse probability weighting were used.

**Results:**

The composite outcome occurred in 32/80 patients (40.0%) in the SGB-based pathway group and 12/80 (15.0%) under routine care (crude risk difference, 25.0 percentage points; 95% CI, 11.7–38.3; crude RR, 2.67; 95% CI, 1.48–4.79). The core robust Poisson model yielded an adjusted RR of 2.43 (95% CI, 1.32–4.46), and marginal standardization yielded adjusted risks of 38.0% vs. 16.7% and an adjusted risk difference of 21.3 percentage points (95% CI, 8.9–32.3). The association was primarily driven by ≥50% operational-burden reduction (33.8% vs. 11.2%; adjusted RR, 2.77; 95% CI, 1.34–5.75). Transition to monotherapy and complete discontinuation were numerically more frequent but imprecisely estimated. Class-specific analyses supported an association with ≥50% benzodiazepine dose reduction; Z-drug analyses were directionally similar but less precise. Three month maintained response remained more frequent after conservative and censoring-weighted analyses. Fifteen of 80 SGB-treated patients had a recorded procedure-related event; no serious event was recorded.

**Conclusion:**

Participation in an SGB-based care pathway was associated with a higher probability of short-term medication reduction, particularly substantial dose reduction, but evidence for complete discontinuation was inconclusive. Because treatment allocation and co-interventions were nonrandomized, the results do not establish an independent causal effect of SGB.

## Introduction

Chronic insomnia is characterized by persistent difficulty initiating or maintaining sleep, early-morning awakening, nonrestorative sleep, and clinically meaningful daytime impairment. Anxiety symptoms and cognitive-emotional hyperarousal frequently coexist with insomnia and may reinforce sleep-related worry, autonomic arousal, safety behaviors, and reliance on hypnotic or sedative medication (Riemann et al., 2017; Morin and Benca, 2012; Levenson et al., 2015; Taylor et al., 2005; Vgontzas et al., 2001; Baglioni et al., 2011; Krystal, 2012).

Benzodiazepines, non-benzodiazepine Z-drugs, sedating antidepressants, and low-dose antipsychotics are used in routine practice for sleep initiation or maintenance. Although short-term symptom relief may occur, sustained use can be accompanied by tolerance, dependence, residual sedation, cognitive or psychomotor effects, falls, and difficulty discontinuing treatment (Qaseem et al., 2016; Sateia et al., 2017; Pottie et al., 2018; Morin et al., 2004; Lader, 2011; Tannenbaum et al., 2014). Guidelines recommend cognitive behavioral therapy for insomnia (CBT-I), individualized pharmacotherapy, gradual tapering, patient education, and shared decision-making (Qaseem et al., 2016; Sateia et al., 2017; Pottie et al., 2018; Morin et al., 2004; Lader, 2011; Tannenbaum et al., 2014). Implementation remains difficult when CBT-I access is limited, anxiety complicates tapering, or patients have longstanding medication dependence.

SGB involves injection of local anesthetic around the cervicothoracic sympathetic chain and has been investigated for posttraumatic stress symptoms, anxiety-related symptoms, sleep disturbance, autonomic dysfunction, and postoperative sleep problems (Liu et al., 2025; Li et al., 2025; Wang et al., 2026; Wu et al., 2026; Rahimzadeh et al., 2018; Yang et al., 2023; Rae Olmsted et al., 2020; Mulvaney et al., 2025; Narouze, 2014). Most published studies have emphasized symptom scales rather than medication reduction. The present study therefore evaluated a pragmatic care pathway incorporating standardized SGB and routine clinical follow-up, with hypnotic or sedative medication reduction as the principal outcome.

We compared patients receiving the SGB-based care pathway with contemporaneous routine-care patients and examined the robustness of the association using parsimonious outcome regression, absolute-risk standardization, class-specific medication measures, propensity-score methods, and explicit analyses of missing outcomes and procedure-related safety.

## Materials and methods

### Study design, period, and data sources

This was a single-center, retrospective, observational cohort study conducted at the First Hospital of Hebei Medical University. The source period extended from 1 January 2022 through 31 December 2025; included index dates ranged from 16 February 2022 through 7 October 2025. Data were obtained from electronic medical records, prescribing and medication-order systems, SGB procedure logs, outpatient and inpatient encounters, nursing observation records, symptom-scale records, and traceable follow-up documentation.

The study was designed to estimate an exposure–outcome association rather than develop, validate, update, or evaluate an individual-level diagnostic or prognostic prediction model. Regression and propensity-score methods were used for confounding adjustment and effect estimation. Reporting was therefore guided by the STROBE statement (von Elm et al., 2007); PROBAST+AI and TRIPOD+AI were not applicable.

### Cohort construction and control selection

A structured search identified 420 potentially relevant records: 120 SGB procedure candidates and 300 routine-care candidates. Among the SGB candidates, 40 records were excluded because insomnia duration was < 3 months (*n* = 8), anxiety symptoms were not documented (*n* = 6), baseline hypnotic or sedative use was absent (*n* = 5), a major psychiatric or neurological exclusion was present (*n* = 5), a concurrent strong intervention could not be separated from the exposure (*n* = 4), baseline or 1-month medication data were incomplete (*n* = 7), or the record represented a duplicate or repeated SGB course (*n* = 5). All 80 remaining eligible SGB-treated patients were included.

Among the routine-care candidates, 120 records were excluded because insomnia duration was < 3 months (*n* = 20), anxiety symptoms were not documented (*n* = 18), baseline hypnotic or sedative use was absent (*n* = 14), a major psychiatric or neurological exclusion was present (*n* = 16), a concurrent strong intervention was present (*n* = 10), baseline or 1-month medication data were incomplete (*n* = 22), severe obstructive sleep apnea was uncontrolled (*n* = 8), or the record represented a duplicate or repeated observation period (*n* = 12). The remaining 180 patients formed the eligible control pool. Eighty controls were selected by 1:1 calendar-time risk-set sampling without replacement. Each control index encounter occurred in the same calendar month and within ±14 days of the corresponding SGB index date. When multiple eligible controls were available, selection was random and performed without reference to subsequent medication outcomes. One hundred otherwise eligible controls were not selected.

For SGB-treated patients, the index date was the first session in the eligible SGB course. For controls, it was the qualifying routine-care encounter on which all eligibility criteria were met. Baseline covariates were obtained from the 7 days before through the index date. The 1-month assessment was the evaluable record closest to day 30 within a ±10-day window, and the 3-month assessment was the record closest to day 90 within a ±14-day window.

### Study population

Eligible patients were adults aged ≥18 years with a clinical diagnosis or clear documentation of chronic insomnia or insomnia disorder lasting ≥3 months, documented anxiety symptoms or an anxiety-related diagnosis or abnormal anxiety scale, baseline use of at least one hypnotic or sedative medication, and sufficient baseline and 1-month medication information. Exclusion criteria included psychotic disorder, manic episode of bipolar disorder, delirium, definite organic brain disease, severe cognitive impairment, unreliable follow-up, electroconvulsive or modified electroconvulsive therapy, repetitive transcranial magnetic stimulation, other inseparable strong interventions, uncontrolled severe obstructive sleep apnea, severely incomplete SGB documentation, or duplicate inclusion. Only the first eligible observation period was retained.

### Exposure and standardized SGB procedure

The exposure was participation in a clinical pathway that included SGB during the index treatment period. All 80 SGB-treated patients completed four procedures, yielding 320 procedures. Patients were positioned supine with slight neck extension. Under real-time ultrasound guidance at the C7 level, an in-plane 22-gauge, 50-mm block needle was advanced to the prevertebral fascial plane anterior to the longus colli muscle. After negative aspiration and confirmation of the target plane, 5 mL of 2% lidocaine hydrochloride was injected. Treatment was administered once daily for four consecutive days, with left and right sides alternated between sessions.

Procedures were performed by two clinicians with institutional authorization and specific ultrasound-guided SGB training. Expected local-anesthetic spread and the development of Horner signs were used as indicators of successful sympathetic blockade. Patients were observed for 30 min after each procedure.

### Routine care and co-interventions

Routine care could include medication review, clinician-directed tapering, sleep-hygiene education, CBT-I, other psychotherapy, antidepressant or anxiolytic treatment, acupuncture, and other physical therapies. Baseline co-interventions and treatments initiated or intensified between the index date and the 1-month assessment were recorded separately. No single validated taper-readiness instrument, prespecified dose-reduction threshold, or uniform taper-initiation algorithm was identifiable in the retrospective records; decisions to initiate or intensify dose reduction were made within routine clinical care on the basis of the treating clinician's assessment of symptoms, medication use, tolerability, patient preference, and readiness to taper. Post-index co-interventions, including clinician-directed taper plans, were not included as conventional baseline confounders because they could represent mediators or components of the treatment pathway. They were reported descriptively and addressed in exclusion sensitivity analyses.

### Class-specific medication measurement

Medication use was reconstructed at baseline, 1 month, and 3 months from medication reconciliation and follow-up records. Benzodiazepines were expressed as approximate diazepam-equivalent daily doses using published clinical conversion guidance; these values were treated as approximate rather than exact pharmacological equivalence (Specialist Pharmacy Service, 2026; American Society of Addiction Medicine partner organizations, 2025). Zolpidem, zopiclone, and eszopiclone were analyzed separately in World Health Organization defined daily dose (DDD) units using oral DDDs of 10 mg, 7.5 mg, and 2 mg, respectively (World Health Organization, 2026, Collaborating Centre WHO for Drug Statistics Methodology ATC/DDD Index 2026). DDD is a technical drug-utilization unit and does not imply equal therapeutic potency.

Trazodone, mirtazapine, and quetiapine were retained as actual doses and medication-presence or discontinuation variables; no diazepam-equivalent value was assigned to these drugs in the class-specific analyses. A broader operational medication-burden score was retained only as an exploratory cross-class measure. For that score, benzodiazepine dose, Z-drug DDD units, and prespecified standard-dose units for sedating adjuncts were combined for within-patient change assessment; the score was not labeled or interpreted as a pharmacological diazepam equivalent.

### Outcomes

The pragmatic primary outcome was achievement of at least one clinically relevant medication-reduction milestone at 1 month: (1) a ≥50% reduction in the exploratory operational medication-burden score; (2) transition from combination therapy with at least two hypnotic or sedative medications to monotherapy; or (3) complete discontinuation of hypnotic or sedative medication maintained for ≥2 weeks. The components differed in clinical magnitude and were not mutually exclusive; complete discontinuation also satisfied the ≥50% reduction criterion. Each component was therefore reported separately, and transition to monotherapy was analyzed only among baseline combination-therapy users.

Class-specific secondary outcomes among baseline users of the relevant class were ≥50% reduction and complete discontinuation of benzodiazepines, and ≥50% reduction in Z-drug DDD units and complete Z-drug discontinuation. Additional outcomes were any medication reduction, 3-month maintained response, symptom changes standardized within the original sleep, anxiety, or depression scale, and procedure-related adverse events.

### Missing outcomes and safety ascertainment

The 1-month medication outcome was available for all participants. Three-month medication information was available for 67/80 SGB-treated patients and 66/80 controls. The primary 3 month analysis used observed follow-up; a conservative analysis classified missing responses as non-maintained, and inverse-probability-of-censoring weighting (IPCW) was used as an additional sensitivity analysis. Sleep, anxiety, and depression change outcomes were available for 142, 144, and 136 participants, respectively. Complete-case analyses were supplemented by inverse-probability-of-observation weighting.

Procedure-related adverse events were ascertained from procedure records and active nursing observation from needle insertion through the 30-min post-procedure period. Delayed events were captured from subsequent clinical documentation through day 7. Event onset, severity, duration, management, resolution, and seriousness were extracted when documented. Horner signs were treated as expected block indicators rather than adverse events.

### Statistical analysis

Continuous variables were summarized as mean (standard deviation) or median (interquartile range), and categorical variables as number (percentage). Baseline balance was evaluated using standardized mean differences (SMDs), with |SMD|≥0.10 indicating potential imbalance. Crude risk differences, risk ratios (RRs), odds ratios (ORs), and 95% confidence intervals (CIs) were calculated.

Because only 44 participants met the primary outcome, the core adjusted model was restricted a priori to treatment group, age, sex, baseline operational medication burden, and baseline number of hypnotic or sedative medications. This model contained five slope parameters, corresponding to 8.8 events per parameter. Logistic regression estimated ORs, robust Poisson regression estimated RRs (Zou, 2004), and Firth penalized logistic regression assessed small-sample stability (Heinze and Schemper, 2002). Variance inflation factors were examined. Model stability was further assessed using 1,000 non-parametric bootstrap samples.

Adjusted risks and absolute risk differences were estimated by marginal standardization, predicting each participant's outcome under both exposure states while retaining the observed covariate distribution. Bootstrap percentile CIs used 1,000 resamples. To examine reviewer-identified imbalances without overfitting, baseline depression severity, antidepressant use, thyroid disease, baseline medication-use frequency, and BMI were each added separately to the core model.

Propensity scores were estimated from verified pretreatment demographic and clinical variables. One-to-one nearest-neighbor matching on the logit propensity score used a caliper of 0.20 standard deviations, without replacement, and targeted the average treatment effect among treated patients (ATT). Overlap weighting used weights 1–e(X) for treated patients and e(X) for controls and targeted the average treatment effect in the overlap population (ATO) (Li et al., 2018). Stabilized inverse probability of treatment weighting targeted the average treatment effect (ATE), with weights truncated at the 1^st^ and 99^th^ percentiles. Robust sandwich standard errors were used. Full SMDs, variance ratios, propensity-score overlap, weight distributions, and effective sample sizes were examined.

Sensitivity analyses excluded controls crossing to SGB within 3 months, patients receiving baseline antidepressant or anxiolytic treatment, and patients receiving CBT-I or psychotherapy. Two-sided P < 0.05 was considered statistically significant. Estimates are interpreted as associations, not causal effects. Analyses were performed in R.

### Ethics

This retrospective observational study was approved by the Clinical Research Ethics Review Committee of the First Hospital of Hebei Medical University (Approval No. [2026] Research Ethics Review No. [Y008]; Chinese approval number: [2026]研审第(Y008)号). The requirement for informed consent was waived because the study used de-identified retrospective clinical data. Exported data contained only research identification numbers.

## Results

### Cohort construction, baseline characteristics, and co-interventions

Of 420 potentially relevant records, 160 patients were included: 80 in the SGB-based care pathway and 80 in the contemporaneous routine-care group (Figure 1). All participants had an evaluable 1-month medication outcome. Three-month follow-up was available for 67/80 (83.8%) SGB-treated patients and 66/80 (82.5%) controls.

**Figure 1:**
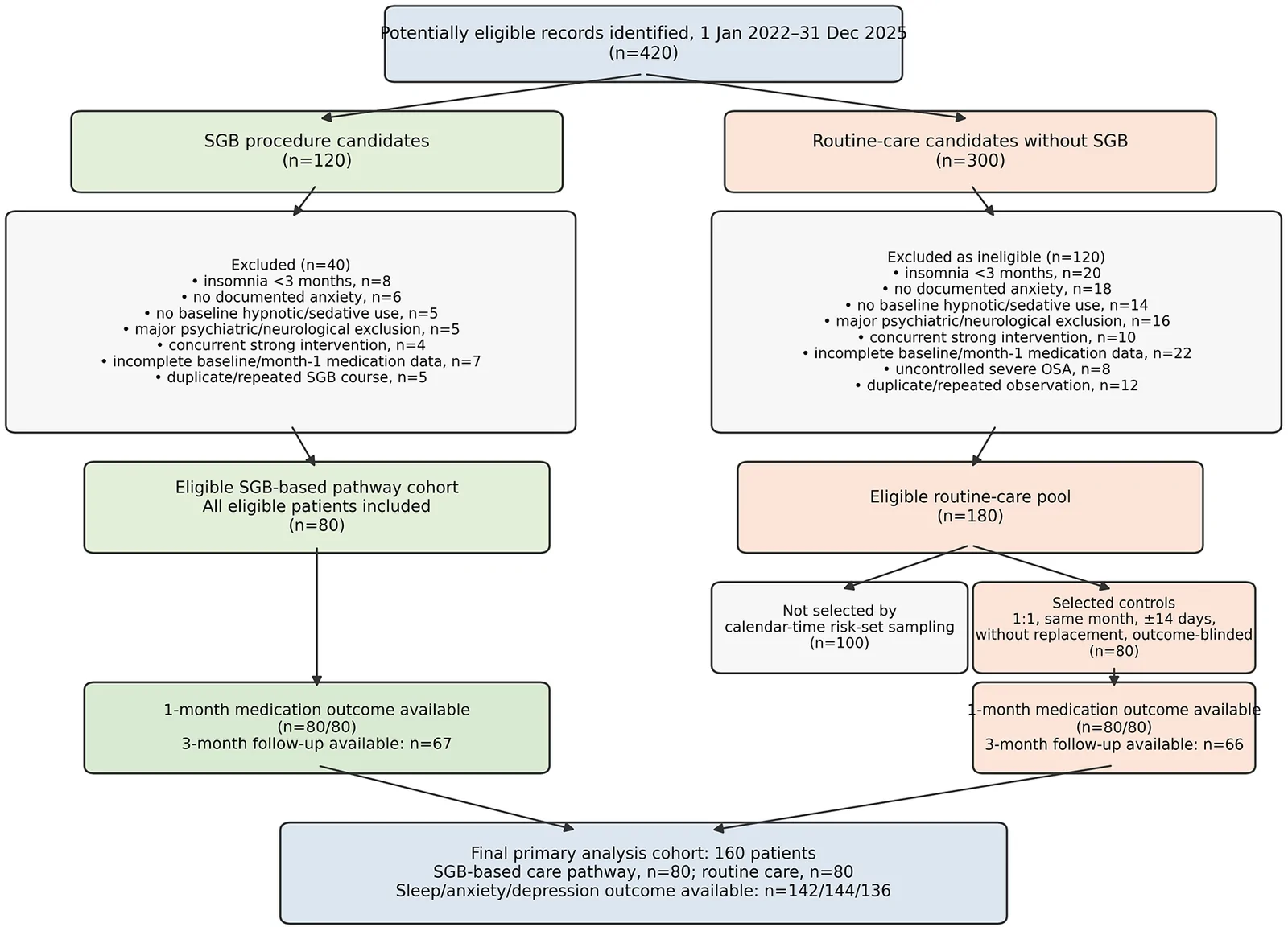
Participant screening, control selection, and follow-up.

Mean age was 45.21 (12.43) years in the SGB group and 44.81 (11.47) years in controls; 61.3% and 65.0%, respectively, were women. Important baseline imbalances included depression severity (SMD, 0.670), antidepressant use (47.5% vs. 22.5%; SMD, 0.543), thyroid disease (5.0% vs 17.5%; SMD, 0.404), baseline operational medication burden (12.09 vs. 13.62 units; SMD, 0.290), medication-use frequency (5.44 vs. 5.11 days/week; SMD, 0.291), and BMI (24.52 vs. 23.86 kg/m^2^; SMD, 0.177) (Table 1).

**Table 1:** Baseline characteristics of the study population.

Characteristic	Routine care (*n* = 80)	SGB-based pathway (*n* = 80)	SMD
Age, years	44.81 (11.47)	45.21 (12.43)	0.033
Female sex	52 (65.0)	49 (61.3)	0.078
BMI, kg/m^2^	23.86 (3.59)	24.52 (3.86)	0.177
Insomnia duration, months	16.88 (16.44)	14.60 (8.77)	0.173
Baseline operational medication burden	13.62 (5.54)	12.09 (4.99)	0.290
Baseline medication count	1.54 (0.65)	1.62 (0.70)	0.129
Medication-use days/week	5.11 (1.15)	5.44 (1.09)	0.291
Baseline sleep severity, z score	0.05 (0.90)	−0.05 (1.09)	0.101
Baseline anxiety severity, z score	−0.01 (1.09)	0.01 (0.90)	0.018
Baseline depression severity, z score	−0.32 (1.03)	0.32 (0.86)	0.670
Use of ≥2 medications	36 (45.0)	40 (50.0)	0.100
Comorbid depression	25 (31.2)	21 (26.2)	0.111
Thyroid disease	14 (17.5)	4 (5.0)	0.404
Chronic pain	10 (12.5)	12 (15.0)	0.073
Baseline antidepressant use	18 (22.5)	38 (47.5)	0.543
Baseline anxiolytic use	26 (32.5)	31 (38.8)	0.131
Baseline CBT-I	21 (26.2)	24 (30.0)	0.083

*Values are mean (SD) or n (%). The operational medication-burden score is an exploratory cross-class measure, not a pharmacological diazepam equivalent. BMI, body mass index; CBT-I, cognitive behavioral therapy for insomnia; SMD, standardized mean difference*.

Baseline CBT-I was documented in 24 SGB-treated patients and 21 controls, psychotherapy in 20 and 18, and sleep-hygiene education in 60 and 67. Between index and month 1, clinician-directed taper plans were documented in 63 and 55 patients, CBT-I initiation or intensification in 19 and 22, antidepressant dose adjustment in 10 and 12, and anxiolytic dose adjustment in 14 and 13, respectively (Supplementary Table 2). Because taper plans were recorded after the index date and were somewhat more frequent in the SGB-based pathway, they were treated as a post-index co-intervention rather than a baseline confounder.

### Primary composite outcome and adjusted absolute association

The 1-month composite medication-reduction outcome occurred in 32/80 patients (40.0%) in the SGB-based pathway group and 12/80 (15.0%) under routine care. The crude risk difference was 25.0 percentage points (95% CI, 11.7–38.3), the crude RR was 2.67 (95% CI, 1.48–4.79), and the crude OR was 3.78 (95% CI, 1.77–8.07).

In the core parsimonious analysis, the adjusted robust Poisson RR was 2.43 (95% CI, 1.32–4.46; *P* = 0.004). The corresponding logistic OR was 6.21 (95% CI, 2.12–18.20; *P* < 0.001), and the Firth OR was 5.53 (95% CI, 2.06–16.58; *P* < 0.001). Marginal standardization yielded adjusted risks of 38.0% (95% CI, 28.5%−46.8%) under the SGB-based pathway and 16.7% (95% CI, 8.2%−25.7%) under routine care, for an adjusted absolute risk difference of 21.3 percentage points (95% CI, 8.9–32.3) (Table 2).

**Table 2:** Primary outcome models and adjusted absolute association.

Analysis	Effect measure	Estimate (95% CI)	*P* value
Crude comparison	OR	3.78 (1.77–8.07)	< 0.001
Core parsimonious logistic	Adjusted OR	6.21 (2.12–18.20)	< 0.001
Core robust Poisson	Adjusted RR	2.43 (1.32–4.46)	0.004
Core Firth logistic	Adjusted OR	5.53 (2.06–16.58)	< 0.001
Marginal standardized risk: routine care	Risk	16.7% (8.2–25.7)	-
Marginal standardized risk: SGB pathway	Risk	38.0% (28.5–46.8)	-
Marginal standardized difference	Risk difference	21.3 percentage points (8.9–32.3)	-

*The core model included treatment group, age, sex, baseline operational medication burden, and baseline medication count. Forty-four events and five slope parameters yielded 8.8 events per parameter*.

All 1,000 bootstrap repetitions converged. The median bootstrap OR was 6.67, with a wide percentile 95% interval of 2.49–31.12, and the treatment coefficient was positive in 100% of samples. The maximum variance inflation factor was 2.31. Adding baseline depression severity, antidepressant use, thyroid disease, medication-use frequency, or BMI individually changed the log OR by −9.8% to +10.5% without changing its direction.

### Components of the composite outcome

A ≥50% reduction in operational medication burden occurred in 27/80 SGB-treated patients (33.8%) and 9/80 controls (11.2%), corresponding to a crude RR of 3.00 (95% CI, 1.51–5.97) and an adjusted robust RR of 2.77 (95% CI, 1.34–5.75; *P*= 0.006).

Among baseline combination-therapy users, transition to monotherapy occurred in 17/40 patients (42.5%) in the SGB-based pathway and 9/36 (25.0%) under routine care. The Firth OR was 1.96 (95% CI, 0.68–5.97; *P*= 0.217). Complete discontinuation maintained for ≥2 weeks occurred in 8/80 (10.0%) and 3/80 (3.8%), respectively; the Firth OR was 2.47 (95% CI, 0.60–12.19; *P*= 0.214). Thus, the composite association was driven principally by substantial dose reduction, whereas evidence for monotherapy transition and complete discontinuation was inconclusive (Table 3; Figure 2). Relative and absolute association estimates across the principal analytic approaches are summarized in Figure 3.

**Table 3:** Components of the 1-month medication-reduction outcome.

Outcome	SGB pathway	Routine care	Risk difference, pp	RR (95% CI)	OR (95% CI)
Composite: any criterion	32/80 (40.0%)	12/80 (15.0%)	25.0 (11.7-38.3)	2.67 (1.48–4.79)	3.78 (1.77–8.07)
≥50% operational-burden reduction	27/80 (33.8%)	9/80 (11.2%)	22.5 (10.0-35.0)	3.00 (1.51–5.97)	4.02 (1.75–9.25)
Combination to monotherapy^*^	17/40 (42.5%)	9/36 (25.0%)	17.5 (−3.4-38.4)	1.70 (0.87–3.33)	2.22 (0.83–5.91)
Complete discontinuation ≥2 weeks	8/80 (10.0%)	3/80 (3.8%)	6.3 (−1.5-14.0)	2.67 (0.73–9.69)	2.85 (0.73–11.17)

*Components were non-mutually exclusive*. ^*^Restricted to baseline combination-therapy users. pp, percentage points; OR, odds ratio; RR, risk ratio.

**Figure 2:**
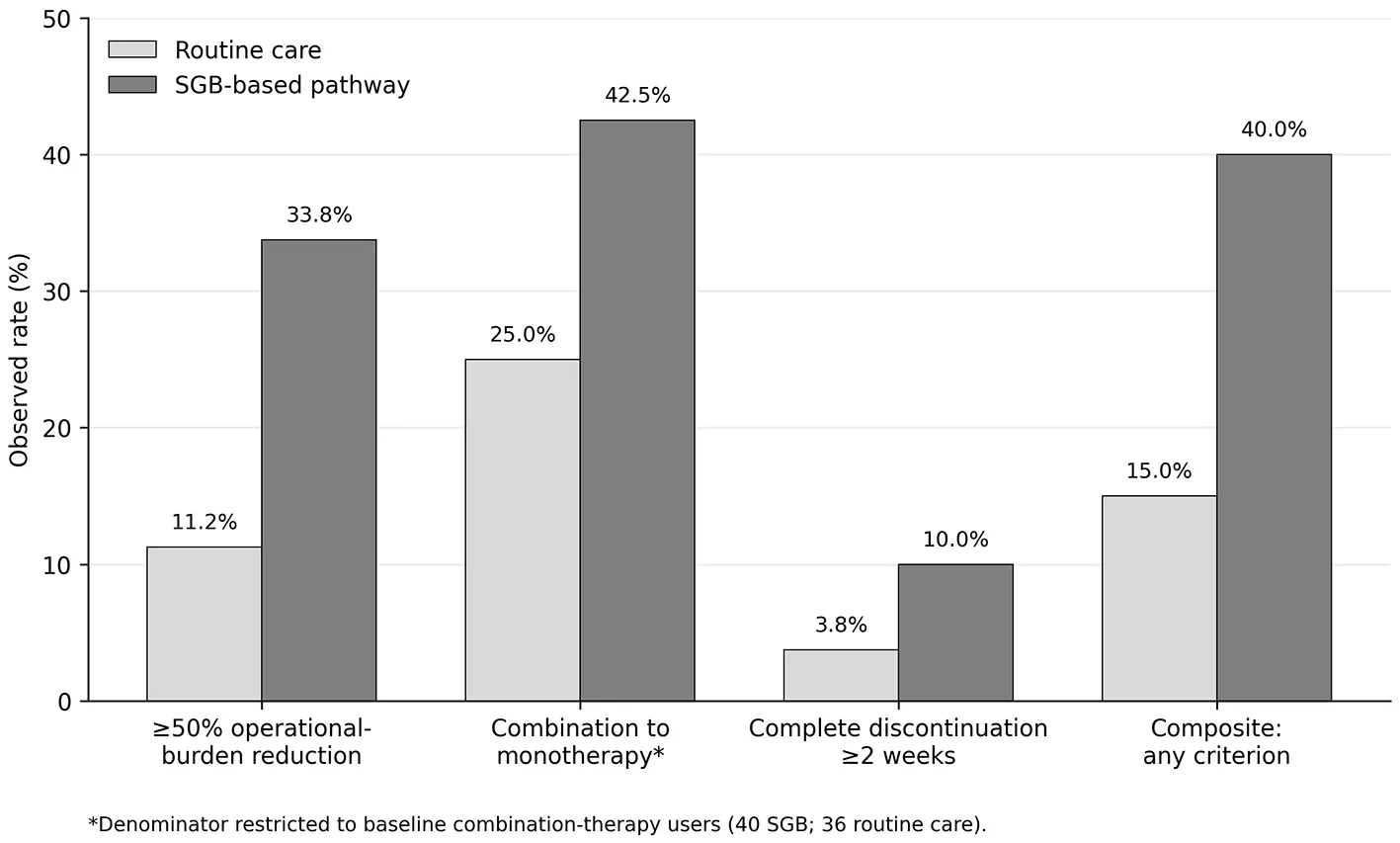
Observed rates for the composite 1-month medication-reduction outcome and its components.

**Figure 3:**
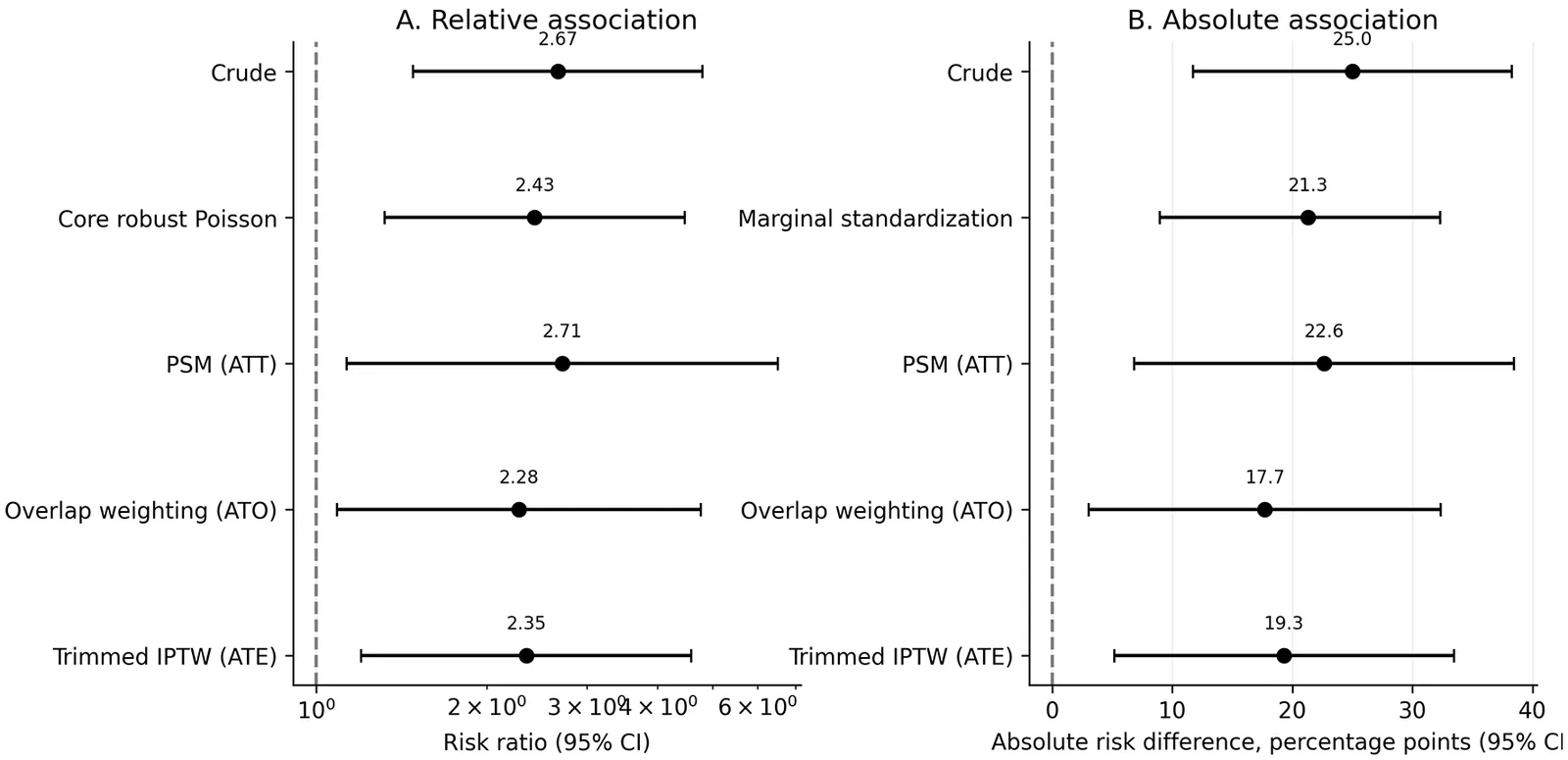
Relative and absolute association estimates for the 1-month primary outcome.

### Class-specific medication analyses

Among 84 baseline benzodiazepine users, ≥50% reduction in benzodiazepine diazepam-equivalent dose occurred in 11/39 SGB-treated patients (28.2%) and 4/45 controls (8.9%). The adjusted robust RR was 3.69 (95% CI, 1.17–11.66; P=0.026), and the Firth OR was 5.25 (95% CI, 1.30–22.49; *P*= 0.012). Complete benzodiazepine discontinuation occurred in 8/39 (20.5%) and 3/45 (6.7%); estimates favored the SGB-based pathway but remained imprecise.

Among 92 baseline Z-drug users, ≥50% reduction in WHO-DDD units occurred in 12/48 patients (25.0%) and 3/44 controls (6.8%). The Firth OR was 4.36 (95% CI, 1.15–21.05; *P*= 0.030), whereas the robust RR was 3.10 (95% CI, 0.81–11.89; *P*= 0.099). Complete Z-drug discontinuation was uncommon and not associated with the care pathway. These analyses support a signal for substantial class-specific dose reduction but not consistent evidence of complete discontinuation (Table 4).

**Table 4:** Class-specific medication-reduction analyses among baseline users.

Outcome	SGB pathway	Routine care	Adjusted robust RR (95% CI)	Adjusted Firth OR (95% CI)
BZD ≥50% reduction	11/39 (28.2%)	4/45 (8.9%)	3.69 (1.17–11.66)	5.25 (1.30–22.49)
BZD complete discontinuation	8/39 (20.5%)	3/45 (6.7%)	2.79 (0.74–10.55)	3.16 (0.83–14.50)
Z-drug ≥50% DDD reduction	12/48 (25.0%)	3/44 (6.8%)	3.10 (0.81–11.89)	4.36 (1.15–21.05)
Z-drug complete discontinuation	5/48 (10.4%)	2/44 (4.5%)	1.06 (0.24–4.60)	1.11 (0.19–7.27)

BZD, benzodiazepine; DDD, defined daily dose. Models adjusted for age, sex, baseline class-specific dose, and baseline medication count.

### Three-month and symptom outcomes

Among patients with observed 3-month follow-up, maintained response occurred in 21/67 SGB-treated patients (31.3%) and 9/66 controls (13.6%; RR, 2.30; 95% CI, 1.14–4.64). When missing follow-up was classified as non-maintained response, rates were 26.3% and 11.3% (RR, 2.33; 95% CI, 1.14–4.78). IPCW produced an RR of 2.29 (95% CI, 1.11–4.72) and a risk difference of 16.5 percentage points (95% CI, 2.9–30.0).

Outcome availability was similar between groups, although participants with observed 3-month follow-up had higher baseline medication burden than those without follow-up. Treatment group was not associated with follow-up availability (OR, 1.24; 95% CI, 0.44–3.53), whereas baseline operational burden showed a modest association with availability (OR, 1.14 per unit; 95% CI, 1.00–1.29).

In adjusted complete-case analyses, the SGB-based pathway was associated with greater standardized improvement in sleep (between-group difference, −0.67 SD; 95% CI, −0.93 to −0.40), anxiety (−0.52 SD; 95% CI, −0.69 to −0.36), and depression (−0.15 SD; 95% CI, −0.28 to −0.01). Observation-weighted estimates were similar. Because symptom scales were heterogeneous and these outcomes were supportive, they should be interpreted cautiously (Table 5; Figure 4).

**Table 5:** Three-month and symptom outcomes.

Outcome	SGB pathway	Routine care	Adjusted association (95% CI)	Additional result
3-month maintained response, observed	21/67 (31.3%)	9/66 (13.6%)	2.30 (1.14–4.64)	17.7 pp (3.9–31.6)
3-month maintained response, missing=no	21/80 (26.3%)	9/80 (11.3%)	2.33 (1.14–4.78)	15.0 pp (3.1–26.9)
3-month maintained response, IPCW	-	-	2.29 (1.11–4.72)	16.5 pp (2.9–30.0)
Sleep symptom change	*n* = 72	*n* = 70	−0.67 SD (−0.93 to −0.40)	P < 0.001
Anxiety symptom change	*n* = 73	*n* = 71	−0.52 SD (−0.69 to −0.36)	P < 0.001
Depression symptom change	*n* = 69	*n* = 67	−0.15 SD (−0.28 to −0.01)	P=0.031

Negative standardized symptom differences indicate greater improvement in the SGB-based pathway. Symptom outcomes were supportive and used complete-case adjusted analyses; observation-weighted estimates were similar. IPCW, inverse probability of censoring weighting.

**Figure 4:**
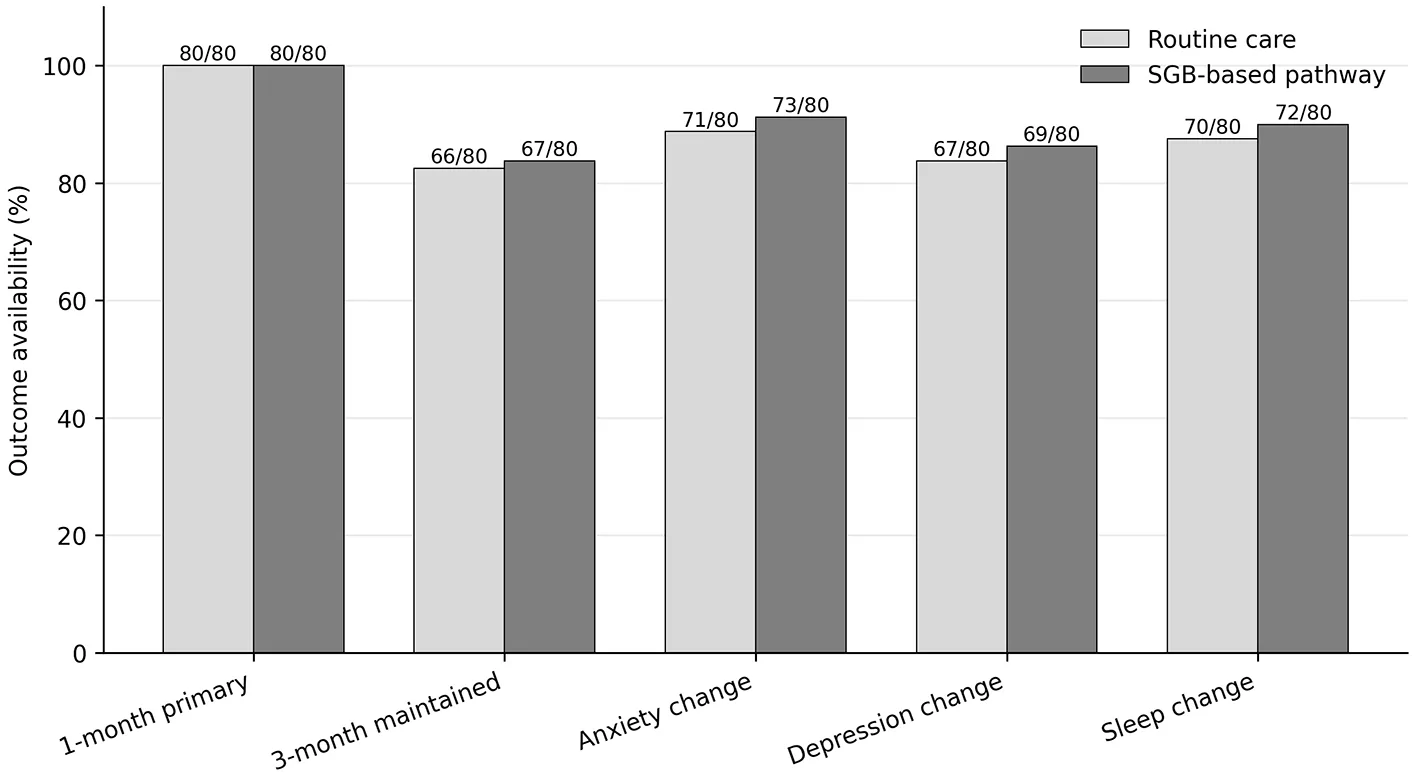
Availability of primary, 3-month, and symptom outcomes by treatment group.

### Propensity-score and sensitivity analyses

Propensity-score distributions overlapped but remained visibly separated, indicating systematic treatment selection (Figure 5). Caliper matching retained 53 pairs. In the matched sample, the outcome occurred in 19/53 SGB-treated patients (35.8%) and 7/53 controls (13.2%); the cluster-robust RR was 2.71 (95% CI, 1.13–6.51), and the risk difference was 22.6 percentage points (95% CI, 6.8–38.4). Residual SMDs >0.10 remained for baseline medication count and baseline sleep severity, and the variance ratio for insomnia duration remained outside the prespecified range.

**Figure 5:**
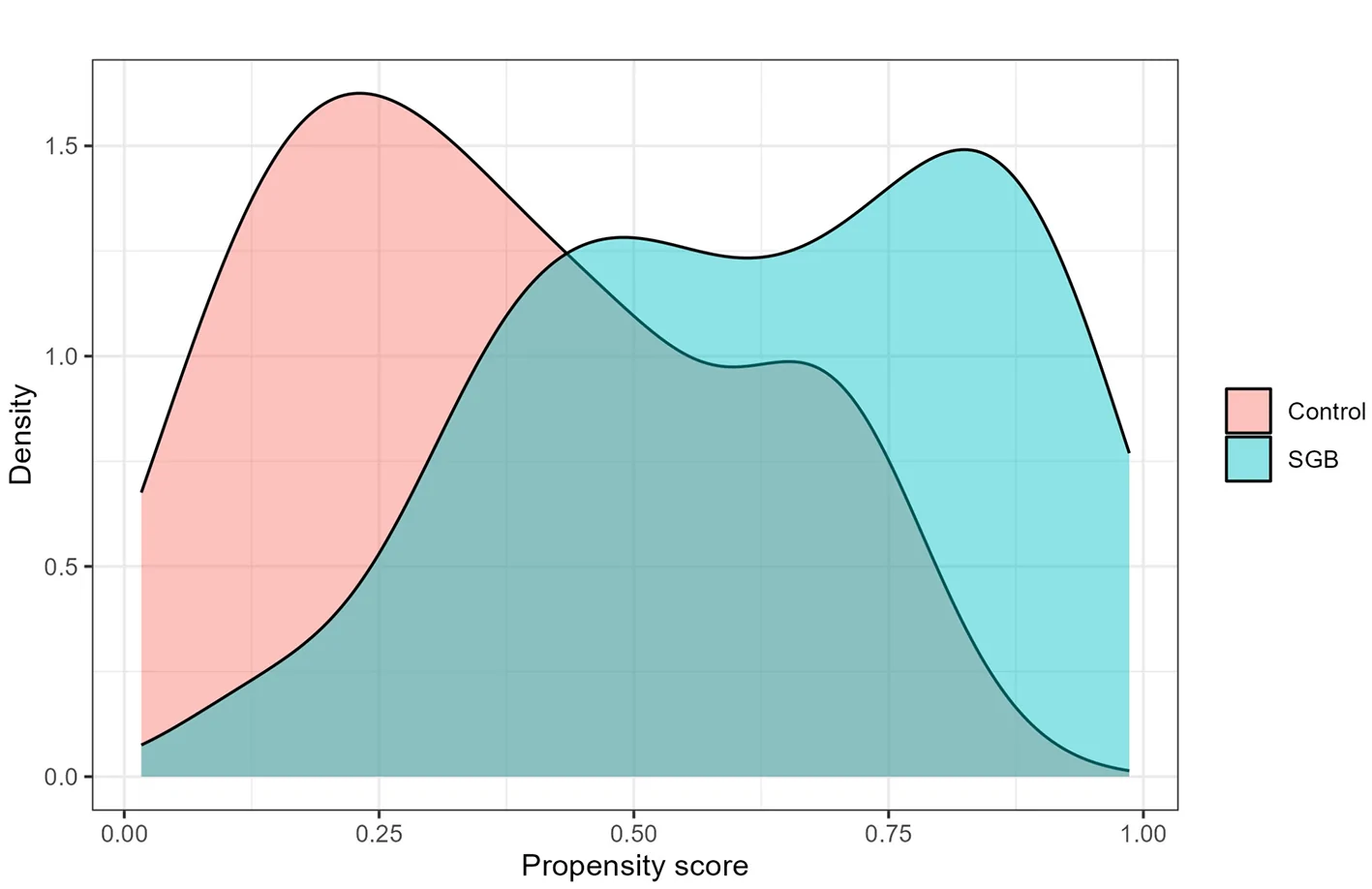
Propensity-score overlap before matching and weighting.

Overlap weighting produced an RR of 2.28 (95% CI, 1.09–4.77) and a risk difference of 17.7 percentage points (95% CI, 3.0–32.4), with an effective sample size of 117.7. Mean balance was near exact by construction, although the variance ratio for insomnia duration remained suboptimal. Trimmed stabilized IPTW produced an RR of 2.35 (95% CI, 1.20–4.58) and a risk difference of 19.3 percentage points (95% CI, 5.2–33.5), with an effective sample size of 123.9. Residual imbalance remained for baseline depression severity and the dispersion of insomnia duration (Table 6; Supplementary Figures S1–S4).

**Table 6:** Propensity-score sensitivity analyses.

Analysis	Estimand	Analysis sample	RR (95% CI)	Risk difference, pp (95% CI)	Balance assessment
PS matching	ATT	53 pairs	2.71 (1.13–6.51)	22.6 (6.8–38.4)	Residual SMDs for medication count and sleep severity
Overlap weighting	ATO	Effective *n* = 117.7	2.28 (1.09–4.77)	17.7 (3.0–32.4)	Mean SMDs near zero; insomnia-duration variance ratio suboptimal
Stabilized trimmed IPTW	ATE	Effective *n* = 123.9	2.35 (1.20–4.58)	19.3 (5.2–33.5)	Residual depression-severity SMD and dispersion imbalance

PS matching used 1:1 nearest-neighbor matching on the logit PS, a caliper of 0.20 SD, and no replacement. Overlap weights were bounded and untrimmed. Stabilized IPTW weights were truncated at the 1st and 99th percentiles. ATT, average treatment effect among treated; ATO, average treatment effect in the overlap population; ATE, average treatment effect.

Sensitivity analyses excluding controls who crossed to SGB by month 3, patients receiving antidepressant or anxiolytic therapy, or patients receiving CBT-I or psychotherapy all retained the same direction of association, although confidence intervals widened.

### Procedure characteristics and safety

All 80 SGB-treated patients completed four standardized sessions, yielding 320 procedures. Fifteen patients (18.8%) had at least one recorded procedure-related event: transient dizziness in 5 (6.3%), hoarseness in 5 (6.3%), puncture-site pain in 3 (3.8%), and vasovagal reaction in 2 (2.5%).

Dizziness, hoarseness, and puncture-site pain were classified as mild; vasovagal reactions were moderate. Median documented durations were 15 min for dizziness, 70 min for hoarseness, 71 min for puncture-site pain, and 18.5 min for vasovagal reactions. Dizziness was managed with supine rest and observation; hoarseness with observation and voice rest; puncture-site pain with local compression and observation; and vasovagal reactions with supine positioning, oxygen, and fluids. Events resolved before discharge or later the same day. No serious procedure-related event was recorded (Table 7).

**Table 7:** Standardized SGB procedures and procedure-related adverse events.

Characteristic/ event	Patients or procedures	Severity	Duration, median (IQR)	Management and resolution
Total procedures	320 procedures; 4 per patient (range, 4–4)	-	-	-
Transient dizziness	5 (6.3%)	Mild	15 (13–16) min	Supine rest and observation; resolved before discharge
Hoarseness	5 (6.3%)	Mild	70 (43–81) min	Observation and voice rest; resolved same day
Puncture-site pain	3 (3.8%)	Mild	71 (68–110.5) min	Local compression and observation; resolved same day
Vasovagal reaction	2 (2.5%)	Moderate	18.5 (18.25–18.75) min	Supine positioning, oxygen, and fluids; resolved before discharge
Serious procedure-related event	0	-	-	None recorded

Active ascertainment covered the procedure and 30-min observation period; delayed events documented through day 7 were also reviewed. Horner signs were expected block indicators and were not classified as adverse events.

## Discussion

### Principal findings

In this retrospective cohort, participation in an SGB-based care pathway was associated with a higher probability of achieving at least one medication-reduction milestone at 1 month. The association was consistent across parsimonious robust Poisson and Firth models, marginal standardization, matching, overlap weighting, and trimmed IPTW. Absolute-risk estimates suggested approximately 18–23 additional medication-reduction outcomes per 100 patients, depending on the target population and adjustment method.

The component analyses materially refined interpretation of the original composite outcome. The strongest and most consistent signal was a ≥50% reduction in medication burden. Transition from combination therapy to monotherapy and complete discontinuation were numerically more frequent but had wide CIs spanning the null. Therefore, the study supports an association with substantial dose reduction rather than evidence that the pathway reliably produces complete discontinuation.

### Medication measurement and clinical interpretation

The revised medication framework separates approximate benzodiazepine diazepam equivalents from Z-drug WHO-DDD units and avoids assigning pharmacological equivalents to trazodone, mirtazapine, or quetiapine. The broader operational score remains useful for within-patient pragmatic monitoring across heterogeneous regimens, but it is not a validated pharmacological scale and should not be interpreted as a universal diazepam-equivalent dose.

Class-specific analyses were directionally compatible with the main result, particularly for ≥50% benzodiazepine reduction. Z-drug reduction also favored the SGB-based pathway, although the RR estimate was less precise. Complete discontinuation outcomes were sparse in both classes. These findings reinforce the need to prioritize continuous or threshold-based dose reduction and durable discontinuation as separate outcomes in future studies.

### Robustness, co-interventions, and causal limitations

Restricting the core model was necessary because only 44 primary events were observed. The resulting 8.8 events per parameter, Firth estimates, low VIFs, and bootstrap consistency support directional stability, but the wide bootstrap interval indicates that the magnitude remains uncertain. The large association between baseline medication count and the composite outcome partly reflects the structural opportunity for combination-therapy users to meet the monotherapy component, which may amplify adjusted ORs. RRs and absolute risk differences are therefore more clinically interpretable than ORs.

Propensity-score analyses addressed measured baseline differences but did not emulate randomization. Propensity-score overlap was incomplete, PSM excluded 27 treated patients, and residual distributional imbalance remained after matching and weighting. In addition, clinician-directed tapering, CBT-I, psychotherapy, and medication adjustments occurred within both care pathways. A clinician-directed taper plan between index and month 1 was documented somewhat more often in the SGB-based pathway (63/80, 78.8%) than under routine care (55/80, 68.8%), raising the possibility of differential taper encouragement. Because this was a post-index co-intervention, adjustment for it in the primary model could have introduced overadjustment if taper counseling was influenced by pathway assignment. These co-interventions may have acted as mediators, competing treatments, or markers of treatment intensity. The results should therefore be attributed to an SGB-based care pathway rather than an independent treatment effect of SGB (Austin, 2011; Brookhart et al., 2006; Stuart, 2010).

### Potential mechanisms and safety

Chronic insomnia with anxiety symptoms is often accompanied by sympathetic hyperarousal. SGB may transiently attenuate cervicothoracic sympathetic output, improve autonomic balance, and reduce stress-related somatic arousal. Symptom improvement and the procedural context may also increase confidence in structured medication reduction. These explanations remain hypothetical because autonomic biomarkers and objective sleep architecture were not systematically measured.

The event-level review provided more complete safety characterization than the original extraction. Recorded events were generally mild or moderate and resolved with conservative management, and no serious event was recorded. Nevertheless, retrospective documentation can miss delayed, subtle, or externally managed complications; these data cannot establish procedural safety with the precision of prospective surveillance.

### Limitations

This study has several limitations. First, the single-center retrospective design is susceptible to selection bias, confounding by indication, and limited generalizability. Second, the modest number of outcome events constrained adjustment and produced wide CIs. Third, important baseline differences remained, including depression severity, antidepressant use, thyroid disease, and medication burden. Targeted, propensity-score, and exclusion analyses cannot eliminate unmeasured confounding.

Fourth, the primary composite combined non-equivalent and overlapping medication milestones, although the revised component analyses mitigate this concern. Fifth, the operational cross-class medication score is not a validated pharmacological equivalence measure; class-specific analyses are more defensible but had smaller denominators and sparse events. Sixth, baseline medication count is structurally related to the monotherapy component. Seventh, 3-month follow-up and symptom outcomes were incomplete, and missingness was associated with medication burden. IPCW and observation weighting rely on unverifiable assumptions. Eighth, symptom scales were heterogeneous. Ninth, safety ascertainment depended on clinical documentation and a limited delayed-event window.

Finally, the comparison estimates the association of an SGB-containing clinical pathway, not the independent effect of the procedure. Patient preference, physician recommendation, economic access, motivation to taper, adherence, and undocumented non-prescription medication use may have influenced both pathway selection and outcomes. Clinician encouragement to taper may also have differed between pathways, and no uniform taper-initiation tool was identifiable in the retrospective records. Moreover, treatment expectations were not systematically measured. Receipt of an invasive procedure may increase expectancy, perceived treatment intensity, clinical engagement, or willingness to reduce medication independently of any physiological effect of sympathetic blockade. Accordingly, the present design cannot disentangle the procedural effect of SGB from taper counseling, other co-interventions, motivation, or expectancy-related effects.

### Future directions

Prospective multicenter studies should use standardized eligibility criteria, randomized or strong quasi-experimental allocation, explicit prespecified criteria for initiating dose reduction, uniform de-prescribing protocols, medication-class-specific dose measures, prespecified complete-discontinuation durability, objective sleep outcomes, systematic co-intervention recording, autonomic biomarkers, and active adverse-event surveillance. Treatment expectations and motivation to taper should be measured prospectively, and, where feasible, sham-controlled or otherwise expectancy-controlled designs should be used to better distinguish procedure-specific effects from non-specific treatment effects. Sample sizes should be adequate for separate benzodiazepine and Z-drug outcomes and for clinically relevant absolute-risk estimation.

## Conclusion

In this single-center retrospective cohort, an SGB-based care pathway was associated with a higher probability of 1-month hypnotic or sedative medication reduction than contemporaneous routine care. The association was driven primarily by substantial dose reduction; evidence for transition to monotherapy and complete discontinuation remained inconclusive. Consistency across parsimonious, absolute-risk, propensity-score, and missing-data analyses supports further prospective evaluation but does not establish an independent causal effect of SGB.

## Data Availability

The datasets presented in this article are not readily available because the de-identified datasets generated and analyzed during the current study may be made available from the corresponding author upon reasonable request. Access is restricted because the data were retrospectively extracted from clinical medical records and are subject to institutional regulations and ethics requirements. Data sharing will require approval by the First Hospital of Hebei Medical University and/or the relevant ethics committee, and may require a data-use agreement. Only de-identified data necessary for scientific verification will be considered for sharing. The data may not be used for purposes beyond the approved request, redistributed to third parties, or used to attempt patient re-identification. Requests to access the datasets should be directed to Yanyong Wang, 57600267@hebmu.edu.cn.
