# Supplementary material for: Association of a stellate ganglion block-based care pathway with hypnotic medication reduction in chronic insomnia with anxiety symptoms: a real-world retrospective cohort study
Source: Front Sleep. 2026 Sep 25;5:1923070. doi: 10.3389/frsle.2026.1923070 (PMC13649451; doi:10.3389/frsle.2026.1923070)
Supplement: Supplementary file 1 [file Supplementary_file.docx]

# Supplementary Material

**Supplementary Table S1. Class-specific medication measurement and handling rules.**

| **Medication** | **Class** | **Revised analytical handling** | **Qualification** |
| --- | --- | --- | --- |
| Diazepam | Benzodiazepine | 10 mg = 10 mg approximate DED | Reference drug; approximate analytical standardization |
| Alprazolam | Benzodiazepine | 0.5 mg ≈ 10 mg diazepam | Approximate clinical conversion |
| Clonazepam | Benzodiazepine | 0.5 mg ≈ 10 mg diazepam | Approximate clinical conversion |
| Estazolam | Benzodiazepine | 1 mg ≈ 10 mg diazepam | Approximate clinical conversion; sources may vary |
| Lorazepam | Benzodiazepine | 1 mg ≈ 10 mg diazepam | Approximate clinical conversion |
| Zolpidem | Z-drug | WHO DDD = 10 mg | DDD unit; not pharmacological equivalence |
| Zopiclone | Z-drug | WHO DDD = 7.5 mg | DDD unit; not pharmacological equivalence |
| Eszopiclone | Z-drug | WHO DDD = 2 mg | DDD unit; not pharmacological equivalence |
| Trazodone | Sedating antidepressant | Actual dose/use/discontinuation only | No equivalence assigned in class-specific analyses |
| Mirtazapine | Sedating antidepressant | Actual dose/use/discontinuation only | No equivalence assigned in class-specific analyses |
| Quetiapine | Low-dose antipsychotic adjunct | Actual dose/use/discontinuation only | No equivalence assigned in class-specific analyses |

DED, diazepam-equivalent dose; DDD, defined daily dose. The broader operational burden score was retained only for exploratory within-patient comparison.

**Supplementary Table S2. Recorded co-interventions by period.**

| **Period** | **Intervention** | **Routine care** | **SGB-based pathway** |
| --- | --- | --- | --- |
| Baseline | Antidepressant | 18 (22.5%) | 38 (47.5%) |
| Baseline | Anxiolytic | 26 (32.5%) | 31 (38.8%) |
| Baseline | CBT-I | 21 (26.2%) | 24 (30.0%) |
| Baseline | Psychotherapy | 18 (22.5%) | 20 (25.0%) |
| Baseline | Sleep-hygiene education | 67 (83.8%) | 60 (75.0%) |
| Baseline | Acupuncture | 11 (13.8%) | 6 (7.5%) |
| Baseline | Other physical therapy | 8 (10.0%) | 3 (3.8%) |
| Index to month 1 | Clinician-directed taper plan | 55 (68.8%) | 63 (78.8%) |
| Index to month 1 | CBT-I initiated/intensified | 22 (27.5%) | 19 (23.8%) |
| Index to month 1 | Antidepressant dose adjustment | 12 (15.0%) | 10 (12.5%) |
| Index to month 1 | Anxiolytic dose adjustment | 13 (16.3%) | 14 (17.5%) |

Post-index co-interventions were described and addressed in sensitivity analyses but were not treated as conventional baseline confounders.

**Supplementary Table S3. Targeted covariate and exclusion sensitivity analyses.**

| **Analysis** | **Approach** | **SGB-pathway estimate (95% CI)** | **Additional information** |
| --- | --- | --- | --- |
| Baseline depression severity | + separately | OR 5.19 (1.73–15.58) | −9.8% change in log OR |
| Baseline antidepressant use | + separately | OR 6.12 (2.02–18.49) | −0.8% |
| Thyroid disease | + separately | OR 6.79 (2.18–21.18) | +4.9% |
| Medication-use days/week | + separately | OR 6.12 (2.05–18.31) | −0.7% |
| BMI | + separately | OR 7.52 (2.40–23.54) | +10.5% |
| Exclude antidepressant/anxiolytic use | Firth sensitivity | OR 5.56 (1.25–32.14) | n=70 |
| Exclude CBT-I/psychotherapy | Firth sensitivity | OR 6.05 (1.72–25.86) | n=92 |
| Exclude control crossover to SGB | Firth sensitivity | OR 4.81 (1.77–14.49) | n=155 |

Each reviewer-identified imbalance was added separately to avoid overparameterizing the 44-event primary model.

**Supplementary Table S4. Summary of propensity-score balance diagnostics.**

| **Analysis** | **Sample** | **Mean-balance summary** | **Residual distributional imbalance** |
| --- | --- | --- | --- |
| PSM (ATT) | 53 pairs | Maximum post-match mean SMD 0.162 | Medication count and sleep severity >0.10; insomnia-duration variance ratio 0.45 |
| Overlap weighting (ATO) | Effective n=117.7 | Mean SMDs approximately 0 | Insomnia-duration variance ratio 0.37 |
| Trimmed IPTW (ATE) | Effective n=123.9 | Depression severity SMD 0.155 | Insomnia-duration variance ratio 0.34 |

Full covariate-specific SMD and variance-ratio diagnostics are represented in Supplementary Figures S1–S3.

**Supplementary Figure S1.** Covariate balance before and after propensity-score matching.

*Alt text: Love plot of standardized mean differences before and after one-to-one propensity-score matching.*

**Supplementary Figure S2.** Covariate balance before and after overlap weighting.

*Alt text: Love plot showing near-zero post-weighting mean standardized differences after overlap weighting.*

**Supplementary Figure S3.** Covariate balance before and after stabilized trimmed inverse probability weighting.

*Alt text: Love plot showing improved covariate balance after stabilized trimmed inverse probability weighting, with residual imbalance in depression severity.*

**Supplementary Figure S4.** Propensity-score weight distributions.

*Alt text: Faceted histograms of raw stabilized inverse probability weights, trimmed stabilized weights, and overlap weights by treatment group.*
